# The Efficacy of Omega-3 Fatty Acid Supplementation for Major Depressive Disorder in Adults: A Systematic Review and Meta-Analysis

**DOI:** 10.1192/bjo.2025.10147

**Published:** 2025-06-20

**Authors:** Oluwatobi Idowu, Nicholas Aderinto, Gbolahan Olatunji, Emmanuel Kokori

**Affiliations:** 1 Cheshire and Wirral Partnership NHS Foundation Trust, Chester, United Kingdom; 2 Department of Medicine, Ladoke Akintola University of Technology, Ogbomoso, Nigeria; 3 Department of Medicine and Surgery, University of Ilorin, Ilorin, Nigeria

## Abstract

**Aims:** Major Depressive Disorder (MDD) is a leading global health concern, significantly impacting quality of life. Conventional treatments like antidepressants are effective but not universally successful, prompting interest in adjunctive therapies. Omega-3 fatty acids, particularly eicosapentaenoic acid (EPA) and docosahexaenoic acid (DHA), are thought to offer neuroprotective and anti-inflammatory benefits that may help manage MDD. This systematic review and meta-analysis assessed the efficacy of omega-3 supplementation in reducing depressive symptoms, improving remission rates, and enhancing overall treatment outcomes in adults with MDD.

**Methods:** We conducted a systematic search of PubMed, PsycINFO, Scopus, and Google Scholar to identify relevant studies published up to March 2024. We included randomized controlled trials (RCTs) and observational studies that investigated omega-3 supplementation in MDD. Studies were selected based on their focus on depressive symptom reduction, remission rates, or relapse prevention. Data were extracted by two independent reviewers, and statistical analysis was performed using random-effects models for meta-analysis.

**Results:** Twenty studies, including 15 RCTs and 5 observational studies with 2,300 participants, met inclusion criteria. Omega-3 supplementation significantly reduced depressive symptoms compared with placebo, with a pooled effect size of Hedge’s g = −0.45 (p<0.01). The most pronounced effects were observed in individuals with moderate-to-severe depressive symptoms. Subgroup analysis revealed that EPA supplementation was more effective when combined with antidepressants. Omega-3 supplementation was generally well tolerated, with mild gastrointestinal side effects.

**Conclusion:** Omega-3 supplementation, particularly EPA and DHA, is effective in reducing depressive symptoms in adults with MDD. Its favourable safety profile makes it a promising adjunctive treatment, especially for patients who do not respond fully to antidepressants. Further research is needed to optimize dosage and identify patient characteristics that predict the best outcomes.

